# An empirical study using permutation-based resampling in meta-regression

**DOI:** 10.1186/2046-4053-1-18

**Published:** 2012-02-23

**Authors:** Joel J Gagnier, David Moher, Heather Boon, Claire Bombardier, Joseph Beyene

**Affiliations:** 1 Department of Orthopaedic Surgery, University of Michigan, Ann Arbor, MI, USA; 2 Department of Epidemiology, University of Michigan, Ann Arbor, MI, USA; 3 Clinical Epidemiology Program, Ottawa Health Research Institute, Ottawa, Ontario, Canada; 4 Department of Epidemiology & Community Medicine, Faculty of Medicine, University of Ottawa, Ottawa, Ontario, Canada; 5 Leslie Dan Faculty of Pharmacy, University of Toronto, Toronto, Ontario, Canada; 6 Health-Policy Management and Evaluation, Faculty of Medicine, University of Toronto, Toronto, Ontario, Canada; 7 Clinical Epidemiology and Biostatistics, McMaster University, Hamilton, Ontario, Canada

## Abstract

**Background:**

In meta-regression, as the number of trials in the analyses decreases, the risk of false positives or false negatives increases. This is partly due to the assumption of normality that may not hold in small samples. Creation of a distribution from the observed trials using permutation methods to calculate *P *values may allow for less spurious findings. Permutation has not been empirically tested in meta-regression. The objective of this study was to perform an empirical investigation to explore the differences in results for meta-analyses on a small number of trials using standard large sample approaches verses permutation-based methods for meta-regression.

**Methods:**

We isolated a sample of randomized controlled clinical trials (RCTs) for interventions that have a small number of trials (herbal medicine trials). Trials were then grouped by herbal species and condition and assessed for methodological quality using the Jadad scale, and data were extracted for each outcome. Finally, we performed meta-analyses on the primary outcome of each group of trials and meta-regression for methodological quality subgroups within each meta-analysis. We used large sample methods and permutation methods in our meta-regression modeling. We then compared final models and final *P *values between methods.

**Results:**

We collected 110 trials across 5 intervention/outcome pairings and 5 to 10 trials per covariate. When applying large sample methods and permutation-based methods in our backwards stepwise regression the covariates in the final models were identical in all cases. The *P *values for the covariates in the final model were larger in 78% (7/9) of the cases for permutation and identical for 22% (2/9) of the cases.

**Conclusions:**

We present empirical evidence that permutation-based resampling may not change final models when using backwards stepwise regression, but may increase *P *values in meta-regression of multiple covariates for relatively small amount of trials.

## Introduction

Systematic reviews are prone to various forms of heterogeneity between included studies. Variability in the participants, interventions and outcomes across studies may be termed clinical heterogeneity; variability in the trial design and quality is typically termed methodological heterogeneity; variability in treatment effects between trials can be termed statistical heterogeneity [1,2]. Methodological heterogeneity hinges on the exact methods of the individual trials, and how they differ from each other. That is, trials that do not properly conceal allocation to treatment groups may bias estimates in treatment effect and cause increased variations in effect between studies included systematic reviews [3]. Significant statistical heterogeneity arising from methodological heterogeneity suggests that the studies are not all estimating the same effect due to suffering from different degrees of bias [2]. In the current work, we focus on clinical heterogeneity that arises from differences in participant characteristics (for example, sex, age, baseline disease severity, ethnicity, and so on), types of outcome measurements, and intervention characteristics (for example, dose, duration of treatment, form of intervention and so on).

In systematic reviews that assess heterogeneity, this is typically examined through subgroup analyses or meta-regression. Subgroup analyses involve dividing the complete dataset into smaller subgroups to make comparisons between them. It is suggested that subgroup analyses be preplanned as part of a systematic review protocol, and even then they should be interpreted with caution [2]. Subgroup analyses may be performed for subsets of participants (for example, males and females) or for intervention characteristics (for example, dose or duration of treatment). These analyses may be performed as a means to investigate heterogeneous results, to answer questions concerning patient groups, types of intervention or types of study. However, as more subgroup analyses are performed on a set of trials, the likelihood of finding false positive or false negative results increases [4].

Meta-regression is an extension of subgroup analyses that allows continuous as well categorical variables to be examined and for the investigation of multiple variables of interest, with the exception of comparisons with less than 10 trials [4]. Meta-regression is similar to simple regression in which an outcome variable is predicted relative to the values of one or more explanatory variables. The outcome variable in meta-regression is the effect estimate, and the explanatory variables (that is, potential effect modifiers or covariates) are any characteristics of the study that might influence the effect estimate. The regression coefficient in meta-regression describes how the treatment effect changes with each unit increase in the explanatory variable and the statistical significance of the coefficient is a test of whether there is a linear relationship between the two. These investigations can be misleading for several reasons [1,2].

First, meta-regression involves making observational associations that are subject to bias (for example, aggregation bias) and confounding (for example, resulting from correlation between characteristics). Also, many systematic reviews using this technique include only a small number of studies while any one of a large number of characteristics of these studies could be a cause of heterogeneity [1,2]. That is, with a smaller amount of studies, the likelihood of a statistically significant explanatory variable rises; a number of false-positive findings is more likely than in conventional regression [1,2,4]. For these reasons the use of permutation tests have been suggested to assess the 'true' statistical significance of an observed meta-regression finding for individual covariates [1,2,4].

The permutation test is useful for analyses of a small number of studies or a large number of covariates and has the goal of maintaining the type 1 error rate [4]. Permutation-based resampling tests the hypotheses of no effect when the distribution of the test statistic is unknown or when the data are not randomly sampled from a defined population, as is often the case in primary meta-analyses or meta-regression [5]. For example, if we were testing the effect of allocation concealment on the summary treatment effects, and we want to compare the summary effect of those trials that performed adequate allocation concealment (group A) and those that did not (group B), we would test the equality of the two summary treatment effects (one for A and one for B) to determine if they likely originated from the same sampling distribution. For meta-regression the associated *P *value is based upon the assumption that the two statistics were sampled from a normal distribution. For permutation tests, a covariance matrix, or distribution, is created for all possible pairs of observations for groups A and B (total is 32 possible pairs of observations for 2 groups across 5 pairs of trials, or 32 matrices and a summary treatment effect difference for each). Next, a ratio is created relative to the frequency that the observed difference in treatment effect found in the primary analysis exceed the overall summary differences obtained from the permutation analysis resulting in a *P *value (for example, 3/32; *P *= 0.09). A simulation study has demonstrated that permutation analyses result in more conservative *P *values and less false positive rates than standard meta-regression (when referring to the t statistic) especially in the presence of heterogeneity, when there are a small number of studies, or when there are a large number of covariates [4].

Due to the large number of treatment level covariates in trials of herbal interventions [6] and the small number of clinical trials included in systematic reviews of these interventions [7] it is important to decrease the false positive rate in meta-regression analyses while still providing valuable information on which characteristics of the intervention or other trial characteristics influence treatment effects. This may lead to increasingly valid and clinically relevant systematic reviews. With this in mind, our objective was to explore the differences in meta-regression modeling results for a predetermined set of covariates using standard large sample approaches verses permutation-based resampling methods.

## Methods

### Trial inclusion

From a sample of 406 randomized controlled trials (RCTs) of herbal medicine identified through a literature search reported elsewhere [8], 2 individuals (JJG, HB) with expertise in herbal medicine interventions identified a selection of herbal intervention/condition pairings that would include the largest number of trials for this project. Through consensus they decided on the following pairings: *Hypericum perforatum *L. (St. John's wort) for treating patients with depression, *Ginkgo biloba *(ginkgo) for dementia, *Serenoa repens *(saw palmetto) for benign prostatic hypertrophy, and *Allium sativum *(garlic) for blood lipids (triglycerides, lipoproteins and cholesterol). Next, we examined the details of the included RCTs for each pairing and determined which trials included similar outcome measures. All other trials were excluded from the analyses.

A generally accepted 'rule of thumb' is that ten events per predictor variable (EPV) will maintain bias and variability at acceptable levels. This guidance derives from two simulation studies carried out for logistic and Cox modeling strategies [9-11]. This has been adapted to meta-regression and thus it is suggested that for each covariate there should be at least ten trials to avoid spurious findings [12]. Therefore, we sought to include a minimum of 20 RCTs for each intervention/outcome pairing to allow for valid meta-regression with 2 or more covariates.

### Development of intervention level covariates

Next, we identified a variety of intervention level covariates for which to explore heterogeneity. We consulted a group of four individuals with expertise in RCTs of herbal medicine interventions who were asked to identify characteristics of the herbal medicine interventions that might influence estimates of treatment effect (for example, active constituent levels). The list intervention level covariates was compiled and included: dose, duration of use, percentage of active constituent, part of plant used, form of product (for example, liquid, dried, powder), method of extraction, and the extraction solvent used.

### Methodological quality assessment

Methodological quality was assessed separately and independently by two individuals. We assessed the sequence generation, allocation sequence concealment, blinding, and description of withdrawals and dropouts, which was the Cochrane practice at the time of this research [13,14]. Each question was answered with a yes, no or don't know response option.

All assessments were entered into preformatted extraction forms. The assessors met on a weekly basis to discuss disagreements that were then resolved by consensus. A third party was not required to resolve any disagreements.

### Data extraction

One individual extracted the following data and a second individual checked all extractions: type of intervention, details of the intervention identified above, summary effect estimates (mean differences, odds ratio (OR), relative risk (RR), risk difference (RD), no. needed to treat (NNT), no. needed to harm (NNH)) for the outcomes of interest, baseline values for the outcomes of interest in each group, participant levels characteristics (mean age, proportion of males and females), sample sizes per group (intention to treat where available), measures of central tendency for all groups (mean, median, mode) and measures of variability (SD, SE, range, CI) before and after treatment on all outcomes of interest, trial length (in weeks), and year of publication.

### Statistical analysis

All statistical procedures were performed by one individual (JJG) using STATA 10.0 (Stata, College Station, TX, USA).

### Effect size calculation for individual trials

For each trial, where effect sizes were not reported for continuous outcome measures we calculated them from reported means for all outcomes of interest. For continuous variables the effect sizes calculated were mean differences (MD) from baseline. All effect estimates for the outcomes of interest were calculated on intention to treat (ITT) data where reported. When variance data were not reported as SDs, they were calculated from the trial data using the standard error of the mean (SE), 95% confidence intervals, reported *P *values, ranges or interquartile ranges according to methods described in the Cochrane handbook [14]. When trials did not report information to calculate the variances we imputed values by giving trials the average SD calculated from across the other trials.

### Meta-analysis for overall effect

The main meta-analysis used a random effects model on the weighted mean differences (WMD) of the primary outcome of interest across all trials. We then collected the WMD, together with 95% CI, *P *value for the main effect and I^2 ^results for statistical heterogeneity with the associated *P *value. Where measures of variance were imputed from the average across the other trials, we performed a sensitivity analysis by removing those trials and comparing the results with the findings for the analysis including all trials.

### Exploration of heterogeneity

Statistical heterogeneity was detected with Cochran's Q test [15,16], which tests if the amount of between trial heterogeneity is greater than due to chance and the recently developed I^2 ^statistic measures the magnitude of statistical heterogeneity that can be expected by partitioning out the chance heterogeneity. Therefore, the presence of statistical heterogeneity was tested using Cochran's Q test [15,16] and the magnitude of statistical heterogeneity between trials using I^2 ^[1]. An I^2 ^of 0% to 40% will indicate no observed statistical heterogeneity, 30% to 60% moderate heterogeneity, 50% to 90% substantial heterogeneity, and 75% to 100% considerable heterogeneity [2].

We performed multivariable meta-regression on the summary treatment effect for the covariates identified above, separated into two models for all intervention outcome pairings. Covariates representing similar topics were grouped into models through a small group process with the investigators. The first model (model 1) included methodological covariates (proper randomization, proper double blinding, a description of withdrawal and dropouts (items 2, 4 and 5 of the Jadad scale) and adequate allocation concealment). The second model (model 2) included participant level and intervention level covariates (length of the trial, proportion of males/females, dose of intervention, baseline severity for the intervention/treatment group). All categorical covariates were coded in a binary manner.

We used backwards stepwise elimination in which the variable with the largest non-significant *P *value for contribution to the model was dropped and the regression was repeated. For each model we used standard large sample methods (referring to the t distribution) and permutation-based resampling to arrive at *P *values. Variables were dropped until only significant predictors of the treatment effect remained. To maintain model validity, if a binary coded categorical variable was statistically significant, both categories of the variable were kept in the model. Collinear variables were also dropped from the model and explored individually for significance. Statistically significant *P *values were those less than 0.05. We then compared the final models and models obtained with each method.

In the permutation test we tested the equality of two means: H_o_:u_x _= u_y_. The assumption being if × data are sampled from the distribution Px and the Y data from Py then under the null hypothesis all permutations of observations are equally probable. The test statistic was the WMD from the meta-analysis results above on all levels of the covariates across trials. We then estimated the null distribution for the treatment effect by permuting (randomly shuffling) the observations in the combined × and Y samples to create all possible distributions. We then compared the effect estimate (or difference in effect) obtained with meta-regression for each covariate against the permuted distribution of effects to arrive at a *P *value. All permutations were set at 10,000 shuffles for each analysis and were performed simultaneously across all variables.

## Results

**Figure 1:**
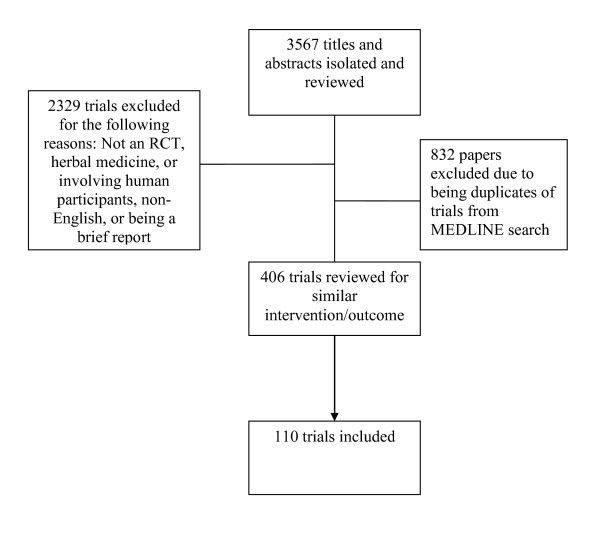
**Flow chart of inclusion of trials**.

A total of 110 trials were included in our analyses (see Figure 1). The intervention outcome pairings are described in Table 1. All other intervention outcome pairings had less than 20 trials and thus were not included. The primary meta-analyses for each of the pairings are listed in Table 2. For all analyses there was a statistically significant effect in favor of the herbal medicine intervention. We also ran a sensitivity analysis removing those trials for which standard deviations were imputed. Results of the sensitivity analyses did not appear to differ from the analyses with all trials included. In the primary analysis there was statistically significant heterogeneity for all analyses with I^2 ^values ranging from 65.7% to 99.1%.

**Table 1:** Description of included intervention condition pairings

Herbal intervention	Outcome measure	Number of RCTs
*Hypericum perforatum *	HAM-D	22
*Allium sativum*	TC	25
*Allium sativum*	LDL	20
*Allium sativum*	HDL	21
*Allium sativum*	TG	22

HAM-D = Hamilton Depression Scale score; HDL = high-density lipoprotein; LDL = low-density lipoprotein; RCT = randomized controlled trial; TC = total cholesterol; TG = triglycerides.

**Table 2:** Results of primary meta-analyses and sensitivity analyses

Intervention outcome pairing	Primary analysis main effect, (95% CI); N trials; I^2 ^(%)	Sensitivity analysis main effect, (95% CI); N trials; I^2 ^(%)
*Hypericum perforatum*-HAM-D	WMD = -3.43 (-4.6 to -2.25); 22^ab^; I^2 ^= 84.7%	WMD = -3.61 (-4.14 to -3.07); 13^ab^; I^2 ^= 87.7%
*Allium sativum*-TC	WMD = -8.95 (-9.65 to -8.25); 25^ab^; I^2 ^= 99.1%	WMD = -9.27 (-9.98 to -8.56); 19^ab^; I^2 ^= 99.3%
*Allium sativum*-LDL	WMD = -4.99 (-5.4 to -4.59); 20^ab^; I^2 ^= 65.7%	WMD = -5.05 (-5.45 to -4.64); 13^ab^; I^2 ^= 96.4%
*Allium sativum*-HDL	WMD = 1.15 (1.05 to 1.25); 21^ab^; I^2 ^= 77.7%	WMD = 1.15 (1.05 to 1.25); 11^ab^; I^2 ^= 86.4%
*Allium sativum*-TG	WMD = -15.78 (-17.30 to -14.24); 22^ab^; I^2 ^= 98.4%	WMD = -15.72 (-17.34 to -14.09); 10^ab^; I^2 ^= 99.2%

^a^*P *< 0.001 for the main effect.

^b^*P *< 0.001 for Cochran's Q test.

HAM-D = Hamilton Depression Scale score; HDL = high-density lipoprotein; LDL = low-density lipoprotein; TC = total cholesterol; TG = triglyceride; WMD = weighted mean differences.

Many of the intervention level covariates were not reported in the included trials. Also, in no instance did a trial report enough information to code a trial as performing inadequate allocation concealment. Therefore, this variable was coded in a binary manner (1 = adequate allocation concealment; 0 = not enough information reported in the trial). For the backward stepwise meta-regression procedures, the covariates remaining in the final models were identical for large sample techniques and for permutation-based resampling methods of arriving at *P *values. Out of a total of ten backwards stepwise procedures, six of the models for both large sample methods and permutation-based resampling resulted in several significant covariates in the final models. Only the models for low-density lipoprotein (LDL)-*A. sativum *(garlic) and for total cholesterol (TC)-*A. sativum *resulted in no covariates remaining significant. The *P *values and I^2 ^of the backwards stepwise elimination procedure for large sample methods (referring to the t table) and the corresponding *P *values using permutation-based resampling including are listed in Tables 3, 4, 5. The *P *values for the covariates in the final model were larger in 78% (7/9) of the cases for permutation and identical for 22% (2/9) of the cases.

**Table 3:** *Hypericum perforatum *HAM-D pairing stepwise elimination results: comparison of final *P *values for final models using both large sample and permutation techniques

Model	Meta-regression (t-distribution), *P *value	Permutation test, *P *value
1. Methodological covariates:		
Appropriate randomization	0.035	0.041
Heterogeneity (I^2^)	81%	
2. Participant level and intervention level covariates:		
Length of trial	0.021	0.022
Sex proportion (male)	0.041	0.045
Heterogeneity (I^2^)	79%	

**Table 4:** *Allium sativum *high-density lipoprotein (HDL) stepwise elimination: comparison of final *P *values for final models using both large sample and permutation techniques

Model 2: participant level and intervention level covariates (N = 19)	Meta-regression (t-distribution), *P *value	Permutation test, *P *value
Mean age of participants	0.003	0.003
Baseline HDL of control group	< 0.001	< 0.001
Heterogeneity (I^2^)	45.8%	

**Table 5:** *Allium sativum *triglyceride (TG) stepwise elimination: comparison of final *P *values for final models using both large sample and permutation techniques

Model	Meta-regression (t-distribution), *P *value	Permutation test, *P *value
1. Methodological covariates (N = 22):		
Appropriate blinding	0.033	0.047
Heterogeneity (I^2^)	98.1%	
2. Participant level and intervention level covariates (N = 17):		
Length of trial	0.001	0.003
Control group TG baseline	0.008	0.016
Dose of garlic	0.031	0.036
Heterogeneity (I^2^)	87.0%	

For the *Hypericum perforatum *(St. John's wort)-Hamilton Depression scale (HAM-D) pairing, the backward stepwise elimination meta-regression and permutation *P *values are listed in Table 3. All *P *values obtained with the permutation tests exceeded those obtained with standard meta-regression. The stepwise elimination meta-regression and permutation *P *values for the influence of covariates on the effect of *Allium sativum *on high-density lipoprotein (HDL) are listed in Table 4. None of the methodological variables reached significance in the first model. In the second model for participant level and intervention level covariates both *P *values obtained with permutation were identical to those obtained with standard large sample meta-regression. This analysis yielded the smallest I^2 ^value (45.8%) of all stepwise elimination values in all models. The stepwise elimination meta-regression and permutation *P *values for the effect of covariates on changes in triglyceride (TG) for *Allium sativum *are listed in Table 5. All *P *values for permutation were larger than those obtained with large sample methods.

In Table 6 we present various features of the final meta-regression models including the number of trials per variable, the magnitude of statistical heterogeneity (I^2^), and the *P *values for both the large sample methods and permutation-based resampling. In the model exhibiting moderate levels of statistical heterogeneity (that is, 45.8%; *A. sativum*-HDL: model 2), and with an adequate number of trials per variable (that is, 9.5) both methods of arriving at *P *values produce identical values.

**Table 6:** Results of final stepwise models relative to number of trials per variable and statistical heterogeneity ranked by number of trials per variable in the final models

Model	TPV	I^2^	Large sample *P *value for the final model	Permutation *P *value for final model
*Allium sativum *TG: model 1	22	98.1%	0.033	0.047
*Hypericum perforatum *HAM-D: model 1	22	81%	0.035	0.041
*Allium sativum *HDL: model 2	9.5	45.8%	0.003	0.003
*Allium sativum *HDL: model 2	9.5	45.8%	< 0.001	< 0.001
*Hypericum perforatum *HAM-D: model 2	8	79%	0.021	0.022
*Hypericum perforatum*HAM-D: model 2	8	79%	0.041	0.045
*Allium sativum *TG: model 2	5.7	87%	0.031	0.036
*Allium sativum *TG: model 2	5.7	87%	0.001	0.003
*Allium sativum *TG: model 2	5.7	87%	0.008	0.016

This table represents the final models for standard large sample techniques, the statistical heterogeneity in the final model, and number of trials included in the final model and the *P *values for both the standard large sample techniques compared to the permutation-based resampling *P *values. Model 1 refers to the stepwise model for methodological covariates. Model 2 refers to the stepwise model for the participant level and intervention level covariates.

HAM-D = Hamilton Depression Scale score; TG = triglyceride; TPV = number of trials per variable (TPV) in each model.

## Discussion

Approximately 50% of systematic reviews use statistical techniques to combine study results and most of these assess consistency across the studies [17]. Several studies report that tests of presence of heterogeneity are frequently performed in meta-analyses, that they are often statistically significant, and that a variety of methods are used to explore heterogeneity, including subgroup analyses and meta-regression [18-21]. Meta-regression may produce spurious findings when performed on a small number of studies, or when investigating multiple covariates [4].

Our study found that using backwards stepwise meta-regression with large sample methods or with permutation-based resampling resulted in identical final models. To the best of our knowledge this is the first empirical test of permutation in meta-regression modeling using an elimination procedure. The results are surprising given that we only had 5 to 6.25 trials per covariate in all of the initial models, which is well below the recommended 10 trials per variable [9-12]. Given that this rule of thumb was derived from simulations for logistic and Cox modeling it is possible that these results do not reliably apply to meta-regression procedures on continuous outcome variables. For example, a simulation study on using linear and logistic regression resulted in a rule of thumb being proposed that, to avoid overfitting when using forward stepwise selection procedures, an event per variable of greater than 4 is required [22-25]. If we extend this observation to linear meta-regression with a backwards stepwise selection procedure then our results seem to make empirical sense. Furthermore, we recommend that more simulations be performed to test the ten trials per variable rule of thumb.

We also found that the *P *values obtained using permutation tests are more conservative, or larger, than *P *values obtained using standard meta-regression methods. Specifically, the *P *values for significant covariates obtained with stepwise meta-regression were larger with permutation 78% of the time and identical 22% of the time compared with using standard large sample methods of obtaining *P *values. This finding is particularly important when *P *values are near a set level of statistical significance (for example, 0.05 or 0.01), since any increase in the *P *value will render the result non-significant. This finding provides empirical evidence that permutation tests increase *P *values and that permutation can be used in meta-analyses especially when examining the effects of multiple covariates, when faced with a relatively small number of included primary studies, or when a large amount of statistical heterogeneity is present. However, these results do not indicate that permutation-based resampling protects against associations arising due to ecological bias. One must always be aware that some associations found at the group level, in this case the study level, may not apply at the individual level.

Our findings are similar to those of Higgins and Thompson [4] who found that when examining multiple covariates in a random effects analysis, permutation resulted in generally larger *P *values, but not in all instances. For example, they found that for the covariate 'intention to treat analysis' the *P *value obtained by meta-regression was larger than that found with permutation. We found that out of nine significant covariates the *P *values for seven of these were larger in the permutation tests. In the other two instances the *P *values were identical. It is possible that the relatively low heterogeneity found in the latter final model (45.8%), or the relative normality of the distribution of effects in the final model resulted in this finding. It is possible that the level of statistical heterogeneity plays a larger role than the number of trials in meta-regression. This hypothesis remains to be tested.

Meta-regression may result in spurious findings with either multiple covariates, with a small number of primary studies [4,25-28] or when there is a large magnitude of statistical heterogeneity. Permutation tests quell *P *values when exploring heterogeneity in these circumstances and will lead to more conservative probability estimates. In some cases the *P *value may cross over to non-significance. This finding is important in meta-analytic research especially since meta-analyses often include a small number of primary studies [4,15], included studies often fail to report important information for these analyses [19], and they often have significant statistical heterogeneity [28-30]. The ability to accurately explore the reasons for heterogeneity and the influence of specific covariates could create increasingly specific and clinically relevant findings in meta-analyses and lead to valid hypothesis generation for future clinical trials. This could potentially save many resources including time, healthcare expenditure, and funding allocation. In agreement with previous recommendations [4] it is advised that permutation tests be used in all meta-analyses that include a small number of clinical trials per covariate (five or less to ten) or that have considerable or substantial heterogeneity.

This study has several strengths. First, we included a large number of primary studies and crosschecked the data extractions. By including a large number of studies we have performed an empirical test of permutation, which goes beyond previous simulations [4]. Next, we used a set of covariates that have empirical and theoretical relationships with our outcome variables. For example, it is well known that adequate randomization sequence generation and allocation concealment are important predictors of effect size [2,3,29,30]. Also, it follows from basic pharmacokinetics that differing doses of the active herbal medicine or its active constituent predict clinical responses [10]. An additional strength of this study was that we ran 10,000 iterations in the permutation tests. Given that permutation tests are random processes, a larger number of permutations results in very similar findings with each additional permutation using the same variable. Therefore, our permutation test *P *values are likely robust. Another strength of this study is that it extends the statistical techniques that can be used in meta-analyses, giving reviewers an empirically validated method when analyzing the influence of covariates.

A drawback of this study is that it may not include all RCTs examining the herbal medicine outcome pairings chosen. That is, the treatment effect estimates presented in Table 2 may not be 'true' estimates of the effect across all published trials. We did not set out to perform a comprehensive meta-analysis on each pairing or to describe the actual or true extent/degree of bias or influence for certain covariates on summary effect estimates. Our objective was to explore the difference in *P *values obtained from standard meta-regression and permutation tests on a sample of trials for several covariates. This project was in effect an empirical exercise comparing two separate statistical techniques for arriving at *P *values. Another potential drawback of this study was that at no point did the permutation test change a significant *P *value to a non-significant one. Even though we did not see this, this would be expected since in most instances the permutation *P *value exceeded the meta-regression values. It is conceivable that such *P *value increases would result in a marginally significant *P *value obtained with meta-regression to become non-significant with permutation [4]. Also, though *P *values are often used to determine the existence of covariate-based effect modification, one should be certain to complement *P *values with actual differences in the effects estimates to determine if changes in the effect are clinically significant. Further research could build upon the intersection of statistical and clinical significance in effect modification. Finally, the last drawback of this research was that data from eight trials for the SJW depression pairing were extracted from Cochrane reviews [31,32]. Therefore, any errors in extraction in the Cochrane review will result in errors in our extractions and the resulting data analyses we present.

## Conclusions

In summary, given that systematic reviews frequently contain a small number of studies and often wish to explore the influence of covariates to explain heterogeneity, the permutation test may help to protect against spurious findings when using meta-regression. However, the changes in significance level we found for the permutation test in the sample of trials we included were small. Furthermore, the relationship between the magnitude of statistical heterogeneity, events per variable and meta-regression with permutation-based resampling should be explored in future research.

## Competing interests

The authors declare that they have no competing interests.

## Authors' contributions

JJG conceived of the study design, performed all data extractions and data analysis and wrote the manuscript. JB conceived of the study design and helped write and edit the manuscript. DM aided in the study design and helped edit the manuscript. HB aided in the study design and helped edit the manuscript. CB aided in the study design and helped edit the manuscript. All authors read and approved the final manuscript.
